# Climate-Driven Aflatoxin M_1_ Risks in Serbia: Implications for Integrated Food Safety Management Along the Dairy Chain

**DOI:** 10.3390/toxins18020105

**Published:** 2026-02-19

**Authors:** Dragan R. Milićević, Božidar Udovički, Ana Šuša, Andreja Rajković, Jelka Pleadin

**Affiliations:** 1 Institute of Meat Hygiene and Technology, Kaćanskog 13, 11000 Belgrade, Serbia; 2 Department of Food Safety and Quality Management, Faculty of Agriculture, University of Belgrade, Nemanjina 6, 11080 Zemun, Belgrade, Serbia; bozidar.udovicki@agrif.bg.ac.rs (B.U.); andreja.rajkovic@ugent.be (A.R.); 3 Institute of Hygiene and Medical Ecology, Faculty of Medicine, University of Belgrade, Dr Subotica 8, 11000 Belgrade, Serbia; ana.s.jovanovic@med.bg.ac.rs; 4 Laboratory of Food Microbiology and Food Preservation, Department of Food Technology, Safety and Health, Faculty of Bioscience Engineering, Ghent University, Coupure Links 635, 9000 Ghent, Belgium; 5 Laboratory for Analytical Chemistry, Croatian Veterinary Institute, Savska Cesta 143, 10000 Zagreb, Croatia; pleadin@veinst.hr

**Keywords:** aflatoxin M1, milk, food safety, risk ranking, risk prioritisation, predictive modeling, climate change

## Abstract

Aflatoxin M1 (AFM_1_) is a carcinogenic milk contaminant and a persistent food safety concern in Serbia, especially under changing climate conditions that exacerbate contamination risks. This review synthesizes national research conducted between 2012 and 2024, covering more than thirty thousand analyzed milk and dairy samples, to evaluate AFM1 contamination, public health risks, and the need for structured risk ranking and prioritization frameworks recommended by the Food and Agriculture Organization (FAO) and the European Food Safety Authority (EFSA). A systematic analysis of Serbian studies explored AFM_1_ occurrence, dietary exposure, and health risk estimates across population groups. The evidence reveals persistent AFM1 contamination with pronounced seasonal peaks during drought years and winter months, frequently exceeding the EU maximum limit of 0.05 µg/kg. Recent multi-year studies confirm that climate-driven AFB1 contamination in maize and compound feed remains a significant and recurring source of AFM1 in milk, highlighting the necessity of structured risk prioritization frameworks. Exposure assessments highlight children and students as the most vulnerable groups, displaying the highest estimated daily intake. Although current margin of exposure (MOE) values remain within acceptable limits, the persistence of contamination underscores a need for proactive risk management. Adoption of FAO and EFSA risk-ranking methodologies would enhance monitoring efficiency, protect high-risk populations, and support alignment with EU standards. Implementing structured risk prioritization is crucial for strengthening Serbia’s food safety governance, guiding policy decisions, and reducing the health burden of AFM_1_ in the dairy sector.

## 1. Introduction

Mycotoxins such as aflatoxins (AFs) represent a serious food safety concern worldwide. AFs are toxic secondary metabolites produced predominantly by *Aspergillus* fungi (especially *A. flavus* and *A. parasiticus*) that commonly contaminate crops like maize, peanuts, and other feeds [1]. Among these toxins, aflatoxin B_1_ (AFB_1_) is the most potent, classified as a Group 1 carcinogen by the International Agency for Research on Cancer (IARC) [2]. When dairy cattle ingest AFB1-contaminated feed, the toxin is metabolized in the liver to aflatoxin M_1_ (AFM_1_) and excreted into the milk. Although AFM1 is a hydroxylated derivative and less acutely toxic (10%) than AFB_1_, it retains significant genotoxic and hepatotoxic potential [3]. Notably, AFM_1_ is also thermally stable, meaning typical heat treatments (pasteurization or boiling) do not effectively destroy it [4]. This combination of carcinogenicity and thermal resistance makes AFM_1_ contamination of milk a critical food safety issue, particularly given milk’s dietary importance for infants and children. 

The carcinogenicity of AFs, particularly their role in liver cancer, has been well established for more than six decades, with epidemiological and molecular evidence consistently demonstrating that exposure to AFs contributes to the development of hepatocellular carcinoma [5]. Global burden assessments indicate that exposure to AFs accounts for an estimated 5–28% of hepatocellular carcinoma cases, representing over 100,000 new cases annually, with the greatest impact observed in sub-Saharan Africa, Southeast Asia, and China, where high HBV prevalence coincides with widespread dietary contamination [6]. Beyond carcinogenesis, accumulating evidence links chronic AFs exposure to immune suppression [7] and impaired growth in children [8,9]. Because milk and dairy products are widely consumed across all age groups, and children in particular ingest milk frequently relative to their body weight, they are especially vulnerable to the chronic health risks of AFM_1_ exposure [10,11]. Evidence from a global 32-year review shows that AFM_1_ contamination of milk is widespread and recurrent, underscoring its significance as a persistent worldwide food safety challenge and emphasizing the need for strong preventive measures to limit its entry into the dairy food chain [12,13].

In the past two decades, climate changes have introduced prolonged hot and dry periods not typical of Serbia’s earlier climate, thereby creating highly favorable agrometeorological conditions for AFs formation. Early surveillance studies [14] conducted between 2008 and 2012 reported markedly elevated frequencies of *Aspergillus flavus* in maize and other crops during years characterized by heat stress and severe drought, indicating a shift toward agroecological conditions that facilitate AFs production. These early signals culminated in the extreme heat and drought of 2012, which resulted in markedly elevated AFB_1_ concentrations in maize and, consequently, increased AFM_1_ transfer into milk. In early 2013, routine monitoring [15] showed that a substantial proportion (76%) of different types of milk exceeded the EU maximum limit for AFM_1_ (0.05 µg/kg) [16], thereby triggering a major national food safety incident. To mitigate the associated market imbalance and maintain consumer protection, Serbian authorities implemented emergency regulatory measures, temporarily increasing the national AFM_1_ limit from 0.05 to 0.5 µg/kg [17], underscoring both the severity of the incident and the economic pressures facing the dairy sector.

In the years that followed, Serbia continued to face intermittent AFM_1_ contamination events. Droughts in 2015, 2017, and 2021, as well as other extreme weather anomalies, led to further surges in maize infection and milk contamination [18,19,20]. Surveillance data indicate that across a 13-year span (2009–2021), AFs were detected in Serbian maize in 9 years, and in five of those years, between one-quarter and four-fifths of tested maize samples were positive [21]. Each spike in AFM_1_ levels tested the resilience of Serbia’s food safety control system and sometimes prompted temporary loosening or adjustment of regulations. By 2014, the crisis-driven limit of 0.5 µg/kg was lowered to 0.25 µg/kg, which remains Serbia’s national standard for raw milk AFM_1_ to date [22]. However, this 0.25 µg/kg threshold is still five times higher than the EU maximum level (ML) [16], reflecting ongoing challenges in fully aligning with stricter international norms. The period from 2012 to 2024 has thus been marked by heightened vigilance, regulatory adaptations, and public concern in Serbia regarding AFs in the dairy supply [23]. Although some monitoring programs have been implemented, the country has lacked a structured, science-based risk management approach for AFM1. This gap is problematic given AFM1’s resistance to elimination and its health impact on consumers. There is a clear imperative for more proactive and systematic risk mitigation strategies in the Serbian dairy sector [24].

To address complex food safety hazards like AFM_1_, international agencies advocate risk ranking and risk prioritization frameworks as part of risk analysis [25]. Risk ranking is an evidence-based process to evaluate and compare different hazards, ordering them by the magnitude of their public health risk [26]. In practice, scientists assess each hazard’s potential severity of health effects (e.g., genotoxicity, carcinogenicity) and the likelihood of exposure to the population. This yields a ranking of hazards—in this case, indicating how AFM_1_ in milk compares to other foodborne risks—based on its estimated impact and exposure frequency. Building on the ranking, risk prioritization is the step where risk managers decide how and where to act on the identified risks [25]. This involves integrating scientific risk ranking with practical considerations, such as public concern, economic impact, and feasibility of control measures, to identify and prioritize food safety issues requiring urgent action. In this respect, structured prioritization approaches such as those reviewed by Van der Fels-Klerx et al. [27] and Hobé et al. [28] demonstrate how health impact metrics, exposure assessment, and trade relevance can be harmonized into actionable monitoring strategies. These frameworks offer reproducible tools to improve the targeting of food safety interventions and disease prevention globally.

In Serbia’s context, adopting a modern risk ranking and prioritization framework is both important and timely. The recurring AFM_1_ incidents have revealed gaps that mirror broader systemic weaknesses previously noted in official assessments [29], including reactive regulatory responses, incomplete harmonization with international standards, and the absence of continuous contaminant monitoring. There is a growing recognition that Serbia’s food safety governance could greatly benefit from a more proactive, risk-based strategy. Such an approach would systematically identify hazards like AFM_1_ that have the highest public health impact, ensure that they are prominently addressed in surveillance programs, and guide policymakers in aligning regulations with actual risk levels. Recent structured risk-ranking analysis conducted for AFM_1_ in Serbian milk and dairy products [30] confirmed AFM_1_ as a persistently high-priority hazard, thereby underscoring the need to shift from descriptive monitoring to evidence-based prioritization.

The aim of this review is to point out the importance and practical necessity of applying structured risk ranking and prioritization tools to strengthen Serbia’s food safety system, particularly in the dairy sector, in light of the AFM_1_ experience. Specifically, the review: (i) synthesizes national evidence on AFM_1_ occurrence, dietary exposure and risk characterization in Serbia during 2012–2024; (ii) maps this evidence onto internationally recognized risk-ranking methodologies provided by the FAO and EFSA and (iii) derives targeted recommendations on how formal risk ranking and prioritization can be integrated into Serbia’s dairy control programs. The study highlights a path toward more resilient, prevention-oriented food safety management that better protects public health, aligns with international best practices, and restores confidence in Serbia’s milk supply in the long term.

## 2. Data Sources and Conceptual Framework

This review integrates multidisciplinary sources of information to support a structured evaluation of AFs risks, particularly for AFM1, within the dairy supply chain. The conceptual framework is grounded in international risk assessment principles and incorporates national surveillance data, peer-reviewed scientific literature, and globally recognized methodologies [31], with particular emphasis on the FAO and EFSA risk-ranking model and the institutional landscape of Serbia’s food safety system. Special attention is given to the alignment with regulatory frameworks and the integration of both horizontal (across stakeholders) and vertical (along the food chain) dimensions of risk governance. The following sections outline the data selection strategy, methodological approaches, and analytical structure used to guide the risk prioritization of AFM1 in Serbia’s dairy sector.

### 2.1. Data Collection and Selection Criteria

To support the risk prioritization framework, the present analysis draws upon a structured review of peer-reviewed literature and national surveillance reports addressing AFM_1_ contamination in milk in Serbia over the period 2012–2024. The data selection process followed predefined inclusion criteria aimed at ensuring scientific validity, contextual relevance, and methodological transparency. Included studies had to meet the following criteria: (i) published in indexed scientific journals or official national reports; (ii) conducted in Serbia or include Serbia-specific data on AFM1 in milk and dairy products; (iii) provide original data on AFM_1_ concentrations, i.e., estimated daily intake (EDI), margin of exposure (MOE) values, or other risk characterization parameters (e.g., HCC incidence); (iv) use validated analytical methods such as ELISA, HPLC, or LC-MS/MS; (v) apply deterministic or probabilistic models for exposure assessment; (vi) include population-specific data (e.g., age-stratified milk consumption, high-risk groups); and (vii) demonstrate alignment with international risk assessment methodologies. Studies that lacked methodological clarity, did not contain quantifiable results, or addressed unrelated contaminants or regions were excluded. The literature was sourced from databases such as Scopus, Web of Science, PubMed, and national repositories. These data provide a robust foundation for applying internationally recognized risk prioritization frameworks, which are presented in the following section. 

### 2.2. Principles of Risk Ranking and Prioritization

In the following section, we describe the FAO [25] and EFSA [26] frameworks that articulate the key principles underlying food safety risk ranking and prioritization. These principles provide structured guidance for decision-makers on what to monitor, when to monitor it, how to implement effective control measures, and where to target interventions along the dairy supply chain. By defining clear criteria for hazard evaluation and intervention prioritization, the FAO and EFSA frameworks serve as foundational tools for enhancing the resilience and effectiveness of food safety systems [32].

#### 2.2.1. Risk Ranking: What to Monitor

**Figure 1:**
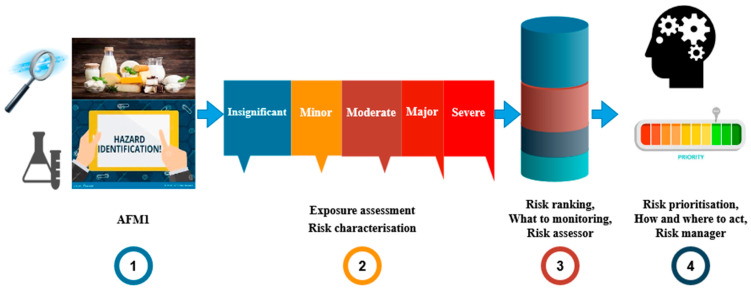
Conceptual flowchart for managing AFM_1_ risk in milk supply chain (adopted by FAO) [25].

Risk ranking is a structured, evidence-based method used to identify which chemical hazards in food present the greatest public health concern [25,26] (Figure 1). It answers the question: “What should we monitor?” by evaluating the severity of health effects (e.g., genotoxicity, carcinogenicity) as well as the probability of exposure, which is primarily influenced by contamination levels, dietary habits, and population vulnerability. This step is especially critical in systems with limited monitoring capacity, as it enables scientific prioritization of hazards for surveillance and control. International bodies like the FAO and EFSA emphasize that ranking should be transparent, reproducible, and rooted in data to support credible decision-making.

#### 2.2.2. Risk Prioritization—How and Where to Act

Risk prioritization builds on ranking to address the next essential question: “How and where should action be taken?” It translates scientific insights into management strategies, integrating additional practical considerations, primarily public and political sensitivity, economic and trade impact, and feasibility of implementation (infrastructure, technology, logistics) [25,26]. The result is a ranked list of actionable interventions, adapted to national circumstances. While based on scientific risk assessments, prioritization also reflects stakeholder values, regulatory feasibility, and capacity for mitigation, making it the bridge between risk assessment and practical food safety management.

### 2.3. Methodologies in Food Safety Risk Ranking: Top-Down and Bottom-Up Approaches

In the context of food safety risk ranking, selecting an appropriate methodological framework is essential. The FAO [25] outlines two complementary approaches: top-down, which begins with population-level public health impact data, and bottom-up, which focuses on hazard occurrence and exposure metrics derived from surveillance and laboratory monitoring. Each approach carries distinct advantages and limitations depending on the availability and quality of data, regulatory goals, and institutional capacity.

**Figure 2:**
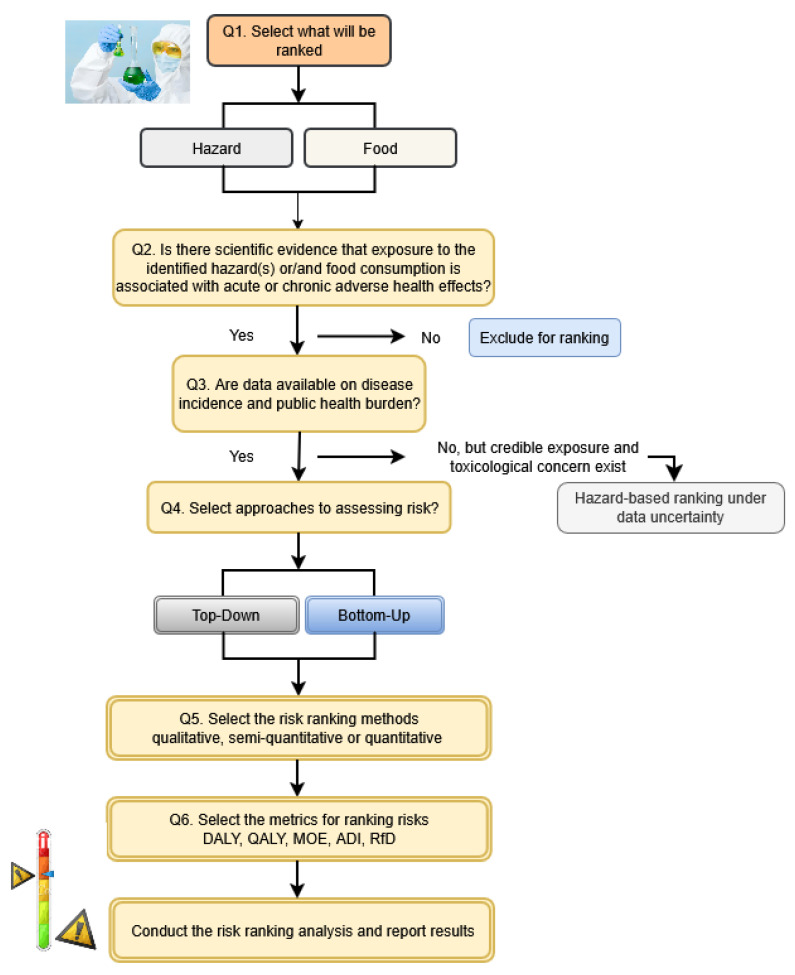
Decision flow for applying risk-ranking principles and methodologies to selected hazards or foods (adopted by FAO) [25].

The following sections describe each approach in more detail, including their respective methodological tools and application in food safety risk ranking. To facilitate understanding of these concepts, Figure 2 provides a visual representation of the decision flow guiding the selection of the most appropriate approach. The diagram outlines the logical sequence from the initial assessment of hazard relevance, through data availability evaluation, to the choice between top-down and bottom-up methodologies and the corresponding risk metrics. These methodological frameworks will later be contextualized in the case study of Serbia, illustrating how top-down and bottom-up approaches can be applied to evaluate and prioritize AFM1 risks in the national dairy sector. 

#### 2.3.1. Top-Down Approaches Based on Public Health Outcomes

The top-down approach begins with an evaluation of foodborne hazards based on their public health impact at the population level. Key metrics used include Disability-Adjusted Life Years (DALYs), Quality-Adjusted Life Years (QALYs), incidence, and mortality rates. This method draws from national or global epidemiological data and burden-of-disease assessments, such as those published by the World Health Organization (WHO) or EFSA. A leading national example is the Danish model [33,34], which used DALYs to prioritize foodborne hazards and guide resource allocation, offering a structured model for public health-driven food policy. In the context of AFM_1_, for instance, a top-down assessment might estimate the attributable fraction of HCC due to chronic exposure through contaminated milk [35]. Where available, such analyses provide a powerful foundation for identifying high-impact hazards that warrant regulatory intervention.

However, this approach requires comprehensive surveillance and disease attribution infrastructure, which may not be available in all countries, particularly for chronic or chemical hazards where direct causal links are difficult to establish. In such contexts, bottom-up approaches offer an alternative or complementary pathway [36].

#### 2.3.2. Bottom-Up Approaches Based on Hazard and Exposure

The bottom-up approach prioritizes food safety hazards by analyzing measurable parameters such as contamination levels, EDI, and toxicological reference values. This approach is exemplified in Norway’s national mycotoxin program, which employed deterministic and probabilistic models to assess exposure and prioritize risk management actions [37]. It is particularly suitable for chemical hazards, such as AFM1, where epidemiological burden data may be scarce or unavailable [38].

This method typically begins with hazard occurrence data from food monitoring systems (e.g., frequency of AFM_1_ detection in milk) and combines it with exposure modeling (e.g., EDI and MOE). Hazards are then ranked based on the likelihood of exposure and severity of health impact, reflecting the FAO’s framework of risk as a function of likelihood × severity. AFM1 in Serbia provides a relevant example: recurrent climate-induced maize contamination leads to elevated AFM_1_ levels in milk, and bottom-up assessment has already been successfully applied using semi-quantitative scoring and MOE-based analysis [30], clearly identifying AFM_1_ as a top-priority chemical hazard that requires targeted mitigation in the dairy sector.

#### 2.3.3. Methodological Tools Used in Bottom-Up Risk Ranking 

**Figure 3:**
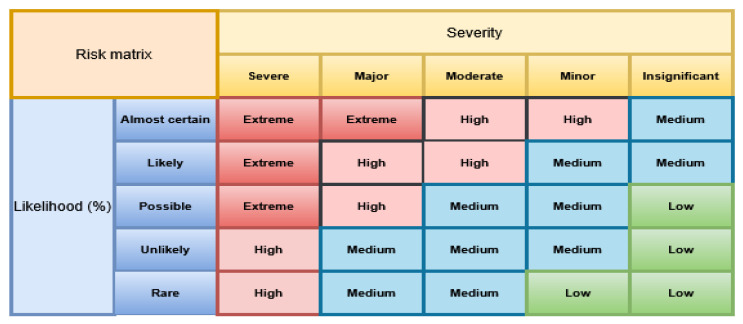
Example of scoring matrix for likelihood and severity (adopted by [36]).

The FAO [25] and EFSA [26] outline complementary categories of methods for bottom-up risk ranking, reflecting their applicability depending on data availability and the specific context of hazard assessment:

(i) Qualitative methods—e.g., decision trees or expert judgment; suitable when data are scarce [36]. A practical application of such qualitative decision-tree approaches is provided later in this review (Section 3.3.1), where a decision tree is used to support risk ranking and prioritization of AFM1 in the dairy chain; 

(ii) Semi-quantitative methods—such as scoring matrices (Figure 3) [36] or Multi-Criteria Decision Analysis (MCDA), integrating toxicological profiles, exposure estimates, trade impact and expert knowledge, were successfully applied in a global prioritization scheme for contaminants in feed and raw materials [28];

(iii) Quantitative methods—rely on measured contamination levels, consumption data, and toxicological thresholds, often using probabilistic models such as Monte Carlo simulations or MOE-based rankings. These approaches have been effectively applied in risk-based monitoring to identify critical control points, particularly in feed chains [27].

In the Risk Matrix methodology, illustrated in Figure 3, the likelihood axis represents the estimated probability that a hazard will occur, with categories ranging from rare, unlikely, possible, likely, and almost certain. These categories are derived from available data, such as the frequency of hazard detection in food samples, historical monitoring results, or expert assessments. The second axis, consequences, reflects the potential impact of the hazard on public health or the food chain, classified as Insignificant, Minor, Moderate, Major, or Severe. By combining these two dimensions, each hazard is placed within a risk category—L (Low), M (Moderate), H (High), or E (Extreme)—which guides prioritization of risk management actions. This visual semi-quantitative approach allows risk managers to easily identify hazards that pose the greatest threat, even when quantitative data are limited. It supports transparent decision-making by clearly showing how the combination of hazard frequency and severity determines the overall risk level. This semi-quantitative matrix approach was also applied in our recent study [30], where hazard likelihood and severity scores were combined to generate a structured ranking of AFM1 risks in the Serbian dairy chain. 

In the case of AFM_1_ in milk, quantitative bottom-up ranking uses MOE values to compare exposure against benchmark dose levels (BMDLs). When MOE < 10,000, this indicates a potential public health concern [38]. Alternatively, semi-quantitative approaches may assign numerical scores based on frequency of detection, contamination levels, and potential health consequences. These tools can also incorporate contextual factors such as geographic origin, seasonality, and population vulnerability. Proper selection and application of these tools ensure that even with limited resources, risk managers can systematically identify and prioritize high-risk hazards.

### 2.4. Risk Ranking and Prioritization: Focus on the Dairy Chain

When determining risk from AFs exposure in the dairy chain, multiple factors must be taken into account. AFB_1_, when ingested through contaminated feed, is rapidly biotransformed in dairy animals, resulting in the presence of AFM_1_ in milk within 24 h. The transfer of AFB_1_ to AFM_1_ in milk, known as the carry-over rate, ranges from 0.1% to 6%, depending on factors such as the animal’s health status, lactation stage, milk yield, feed composition and feasibility of replacing contaminated feed ingredients [39,40,41]. Due to its thermal stability, AFM1 persists through standard processing steps such as pasteurization and UHT treatment, thus remaining in final dairy products including cheese, yogurt, whey and powdered milk [42,43,44,45].

Although AFM_1_ is approximately ten times less potent than AFB_1_ [38], it retains significant genotoxic and carcinogenic properties as a Group 1 human carcinogen [2]). These toxicological characteristics, combined with its persistence and wide dietary distribution, make AFM1 a critical chemical hazard in the milk supply chain. Particular concern is warranted for infants and young children, who consume more milk relative to their body weight and are therefore at greater risk from chronic exposure.

#### 2.4.1. Risk Ranking: Key Inputs and Indicators

Risk ranking of foodborne hazards requires an integrated methodological framework that combines both horizontal and vertical dimensions of analysis. These two layers of integration ensure a comprehensive understanding of the nature, frequency, and severity of risks posed to public health [46].

(i) Horizontal integration is aligned with the four sequential steps of risk assessment, providing a scientific and evidence-based foundation for ranking hazards. This includes toxicological and epidemiological data, contamination dynamics (e.g., carry-over rate), exposure modeling, and risk interpretation.

(ii) Vertical integration introduces structured methodologies to quantify and compare consequences across hazards and/or food commodities, using public health metrics such as DALYs, QALYs, MOE, or semi-quantitative scoring matrices.

- Horizontal Integration: Risk Assessment Framework

The horizontal dimension of risk ranking relies on the structured process of risk assessment, composed of the following steps:

(i) Hazard Identification: AFM_1_ is a hydroxylated metabolite of AFB_1_, formed in the liver of lactating dairy cows after ingestion of contaminated feed. It is excreted in milk and resistant to pasteurization. A key parameter in this context is the Carry-Over Rate (COR), defined as the percentage of ingested AFB1 that is transferred into milk as AFM_1_. Understanding the COR is essential for estimating contamination in milk and downstream human exposure [39,40,41].

(ii) Hazard Characterization: AFM_1_ is classified as a Group 1 human carcinogen by the IARC, confirming its genotoxic and carcinogenic potential [2]. The EFSA establishes a benchmark dose lower confidence limit (BMDL_10_) of 0.4 µg/kg body weight/day for AFB_1_, which serves as a reference point in toxicological risk characterization [38]. In line with health-based guidance values (HBGVs), a potency factor of 0.1 was applied to AFM1, reflecting its lower carcinogenic potency compared to AFB1 [38].

(iii) Exposure Assessment: Human exposure to AFM_1_ is calculated as EDI, based on: concentration of AFM_1_ in milk, average daily milk consumption across population groups, and body weight assumptions for different age classes. This step incorporates data from food monitoring and national dietary surveys, allowing for refined estimates in high-risk populations such as infants and children [10,11].

(iv) Risk Characterization: The potential health impact of AFM_1_ is interpreted through the MOE, which compares the BMDL10 to the EDI. An MOE below 10,000 is considered to indicate a potential public health concern, particularly in sensitive subgroups [38]. This metric allows translation of exposure estimates into actionable risk categories for prioritization. These horizontal indicators form the scientific basis for ranking AFM1 in terms of public health importance and facilitate integration with broader hazard prioritization systems [30].

- Vertical Integration: Public Health Impact Metrics

Vertical integration enables comparative evaluation of hazards and/or foods by quantifying the severity of health outcomes, facilitating prioritization across different types of contaminants and food commodities. Key indicators include DALYs, Hazard Quotients (HQs), risk scoring matrices, and MOE values, interpreted in a cross-hazard and cross-commodity context [25,26]. Among public health impact metrics, DALYs are widely recognized as the most robust and comprehensive indicator for capturing the overall burden of disease, as endorsed by the WHO. DALYs integrate both mortality (Years of Life Lost, YLL) and morbidity (Years Lived with Disability, YLD), making them uniquely suited for comparing health impacts across diverse hazards, including AFs. However, the calculation of DALYs is methodologically demanding—it requires detailed epidemiological data, accurate disability weights, and standardized assumptions regarding life expectancy and disease duration. As highlighted by Chen et al. [47], the ongoing evolution of the DALY framework reflects efforts to improve comparability and validity across regions and over time, yet these refinements also underscore the complexity inherent in its application.

While MOE originates from horizontal risk characterization, it can also serve as a proxy indicator in vertical comparisons, particularly for chemical hazards where outcome-based metrics, such as DALYs, are limited or infeasible. In this context, MOE is not used to assess absolute safety, but rather to rank hazards in terms of proximity to toxic thresholds, allowing decision-makers to compare and prioritize multiple hazards based on their relative risk potential. For example, when assessing multiple foodborne contaminants, a hazard consistently producing lower MOE values across population groups would warrant higher priority in national surveillance or mitigation strategies.

#### 2.4.2. Prioritization of Risk Management Actions

Prioritization of risk management actions requires the alignment of scientific risk estimates with feasible and effective control strategies across the food chain. AFs, particularly AFM_1_ in milk, represent a complex challenge where multiple control points, from feed quality to dairy processing, must be considered [40]. To ensure meaningful and efficient intervention, prioritization must integrate both horizontal and vertical dimensions of risk governance [24].

Horizontally, in line with the EU “Farm to Fork” holistic and integrative strategy, requires coordination between actors responsible for feed safety, milk production, public health surveillance, and regulatory enforcement [32,38,45]. Vertically, it involves mapping the contamination pathway—from maize cultivation and feed production to animal metabolism, milk safety, regulatory gaps, consumer exposure, and population vulnerability [19,21,24,48,49]. Effective implementation of this framework requires not only coordinated action across sectors but also robust laboratory capacities to detect contaminants and risk-based inspection systems to ensure compliance along the food chain [32,46]. This dual-layered integration supports decision-making frameworks that guide control measures where they are most needed and most effective, determining where, when, and how to act [18,25,30].

In the dairy chain, the following inputs and management priorities are essential:

(i) Non-compliance history: Repeated exceedances of applicable nationally or internationally recognized maximum residue limits (MRLs) for AFM1, whether in feed or milk, indicate weaknesses in current control measures and highlight the need for intensified oversight in affected supply chains. Historical non-compliance serves as a practical trigger for enhanced inspections and corrective actions [10].

(ii) Geographic and climatic targeting: Areas prone to drought and regions with intensive maize cultivation frequently demonstrate elevated levels of AFB_1_ contamination in feed, which translates into higher AFM_1_ concentrations in milk. Seasonality also plays a key role, with contamination peaks often occurring in late winter or early spring due to stored feed usage. These patterns justify targeted monitoring in specific regions and time periods [19,21,43].

(iii) Temporal prioritization: Enhanced monitoring and control during high-risk seasons, particularly late summer or winter through early winter [23,42].

(iv) Farm-level profiling: Identify high-risk dairy farms characterized by heavy silage usage, inadequate hygiene, or poor feed management practices [50].

(v) Regulatory misalignment: In some jurisdictions, national MRLs for AFM_1_ in milk may not align with international standards (e.g., those of the EU or Codex Alimentarius). Such misalignment can affect public health protection, limit international trade, and reduce consumer confidence. Harmonizing national thresholds with global benchmarks is often a strategic objective in risk prioritization [18,24,30,51].

(vi) Subgroups such as infants and young children are particularly susceptible to AFM_1_ due to their higher milk consumption relative to body weight [10,24]. Risk assessments often show that these groups may have lower MOE, necessitating special monitoring of milk destined for vulnerable consumers, including school programs and infant formula production.

(vii) Feasibility of control measures: Effective risk prioritization must assess the real-world feasibility of implementing mitigation strategies. This includes evaluating available infrastructure, laboratory capacity, farmer knowledge, and the ability to enforce measures such as hazard analysis and critical control points (HACCP) systems [45], use of mycotoxin binders, or feed testing protocols [19].

(viii) Public and political sensitivity: Food safety crises involving AFs often provoke strong public reactions and media scrutiny. In such contexts, transparent communication, pre-emptive action, and risk-based justification for policy decisions become essential to maintain public trust and political accountability [23].

Recommended actions derived from prioritization may include: enhanced surveillance in high-risk areas, seasonal monitoring programs, implementation of early warning systems, risk-based categorization of farms, focused control for vulnerable groups, feasible mitigation measures, regulatory harmonization roadmap, capacity building and training and transparent risk communication [11,49]. This structured approach ensures that control measures are directed where they are most needed, enabling efficient use of resources and strengthening public health protection within the dairy production chain [46].

## 3. Case Study: Serbia

### 3.1. Food Safety Governance and Institutional Structure

The Republic of Serbia operates a food safety governance system structured across multiple institutional levels, based on the principles of shared responsibility, functional specialization, and risk-based oversight. While Serbia’s food safety control framework is largely aligned with EU food legislation and incorporates risk-based principles, certain systemic gaps remain, including incomplete harmonization of national regulations and the absence of several by-laws essential for contaminant monitoring. Despite ongoing efforts under Chapter 12 of the EU accession process, these issues highlight the need for further regulatory consolidation to ensure consistent public health protection [52]. Within this framework, food safety governance is horizontally distributed across several competent authorities, each responsible for distinct segments of the milk production chain, covering both feed and food safety aspects.

Key stakeholders include:

(i) Food Business Operators (FBOs): Responsible for implementing food safety assurance systems (e.g., HACCP) and ensuring the safety of feed and food at all stages of production, processing, and distribution.

(ii) The Plant Protection Directorate and the Agricultural Inspection Sector (Ministry of Agriculture, Forestry and Water Management): Conduct official controls of food and feed of plant origin across different stages of the supply chain, including maize—one of the primary vectors of AFB1 contamination in dairy feed—with responsibilities divided between preventive controls at the primary production level and market-level controls in independent storage facilities.

(iii) The Veterinary Directorate (Ministry of Agriculture, Forestry and Water Management)): Performs tasks related to animal health protection and veterinary–sanitary control, including the monitoring and official control of feed for animal nutrition and food of animal origin throughout the food chain.

(iv) The Ministry of Health: Supervises public health protection, including monitoring of foodborne illnesses and maintaining surveillance systems relevant to chemical and microbiological hazards, and through sanitary inspection services, contributes to official food control at the retail level within the food safety system.

(v) The Directorate for National Reference Laboratories (DNRL): Provides scientific and technical support to the official control system by developing and harmonizing analytical methods, ensuring quality assurance of laboratory testing, and coordinating the network of authorized laboratories involved in food, feed, plant, and animal health analysis. Horizontal governance defines who is responsible for risk management across sectors, whereas vertical integration defines where and how AF risks propagate along the dairy chain. Therefore, this complex yet structured institutional setup underscores the need for effective inter-agency coordination and deeper vertical integration along the dairy value chain—from primary production (feed) through on-farm practices and raw milk collection to processing and consumer-level exposure. In this context, AFs, particularly AFM_1_ in milk, pose a critical food safety challenge, as their control requires synchronized action across all segments of the national system. The shortcomings identified by the State Audit Institution of the Republic of Serbia [29], including incomplete regulatory harmonization and the maintenance of MLs for AFM_1_ that remain less stringent than those stipulated under EU legislation, reinforce the importance of strengthening coordination and fully operationalizing risk-based mechanisms across competent authorities.

Building on this institutional landscape, the following subsections present national data and scientific evidence related to AFM_1_ occurrence, risk-ranking indicators, exposure estimations, and seasonal trends. These considerations provide the basis for the subsequent analysis of national data on AFM_1_ occurrence, risk-ranking indicators, exposure estimations, and seasonal trends, which collectively support the development of targeted, risk-based interventions.

### 3.2. AFM1 Risk Profile in Serbia 

#### 3.2.1. Occurrence of AFM_1_ in Milk

Since the AF crisis of 2012–2013, Serbia has witnessed a steady intensification of scientific efforts aimed at understanding and mitigating AFM_1_ contamination in milk and dairy products. A growing body of research has consistently identified climate change, characterized by prolonged droughts, elevated temperatures, and erratic rainfall patterns, as the principal driver of AFB_1_ accumulation in maize-based feed [53], which subsequently results in AFM_1_ presence in milk [54,55]. 

The analyzed studies, summarized in the accompanying Table 1, offer comparative insights into sample sizes, contamination frequency, concentration ranges, and rates of regulatory exceedance. While descriptive in nature, the collective significance of these works lies in their broader scientific contributions, which can be categorized into five critical thematic domains: (i) contamination trend monitoring and cause identification, (ii) methodological innovations, (iii) improvement of the food safety system, (iv) contribution to public health, and (v) strengthening of interdisciplinary approaches.

Numerous studies have emphasized the seasonal dynamics of AFM_1_ contamination in Serbia [18,19,42,43,45,48,50,57,58,59,60,61,62], particularly during summer and autumn, as seasons marked by increased climatic stress, such as prolonged droughts and elevated temperatures. These environmental conditions, coupled with inadequate storage practices, create favorable conditions for fungal growth and subsequent AFB1 contamination in feed, ultimately leading to AFM_1_ residues in milk. Comparable seasonal and climatic influences on AFM_1_ contamination have been documented in neighboring countries, including Croatia [63,64,65], Albania [66], Hungary [67], Italy [11,49], Kosovo [68], North Macedonia [69], Romania [70], Turkey [71] and Greece [72]. For example, studies from Croatia (e.g., ref. [63] have reported similar temporal peaks of AFM_1_ in milk, highlighting the broader regional impact of climate change on dairy safety.

These findings collectively reinforce the need to conceptualize AFs risk not as a localized problem, but as a regional and transboundary food safety issue shaped by shared climatic drivers [73]. By contextualizing Serbia’s AFM_1_ contamination patterns within the wider Southeast European landscape, it becomes evident that future risk mitigation efforts should incorporate regional collaboration and climate-informed adaptation strategies. This regional framing also supports Serbia’s alignment with EU-wide food safety policies and provides a basis for harmonizing monitoring systems and early warning mechanisms across borders.

Taken together, these findings highlight that AFM_1_ contamination in Serbia represents a complex and evolving food safety challenge shaped by environmental pressures, infrastructural constraints, and regulatory gaps. Tackling this issue calls for integrated food safety systems that incorporate climate-adaptive surveillance strategies and align with EU standards. Preventive strategies must be grounded in the One Health framework, recognizing the interconnections among human health, animal health, and the environment. Such a multidisciplinary approach enables more effective risk management along the entire dairy chain.

Through this systematic review of national scientific studies, Serbia’s progress in monitoring, understanding, and managing AFM_1_ contamination in milk is clearly documented [30]. These studies offer valuable insights for risk managers and policymakers and serve as a scientific foundation for the implementation of predictive models, digital surveillance tools, and targeted interventions for vulnerable population groups. Continued improvement of the system will require sustained interdisciplinary collaboration, investments in analytical infrastructure, and proactive mitigation of climate-driven risks in the dairy chain.

#### 3.2.2. Dietary Intake and Risk Characterization 

The assessment of dietary exposure to AFM_1_ from milk and dairy products is a fundamental step in characterizing public health risks and establishing priorities within food safety management systems. While contamination data are crucial, their interpretation through structured dietary exposure assessments provides the necessary basis for implementing targeted and evidence-based control measures.

Table 2 provides a detailed overview of exposure and risk assessment studies on AFM_1_ intake in Serbia. Early studies, such as those by Skrbić et al. [15] and Kos et al. [56], pointed to alarmingly high levels of exposure across all age groups in Serbia, particularly among children. Exceedance of health-based guidance values by estimated daily intake (EDI) levels underscores the urgent need for integrated food safety control systems. Despite methodological limitations, such as small sample sizes and short sampling periods, these studies served as a catalyst for subsequent, more systematic investigations.

A study conducted by Torović [58] applied a temporal framework for assessing AFM1 exposure, integrating average contamination data from processed milk with national-level consumption statistics. Findings from the study demonstrated a marked reduction in exposure levels between 2013 and 2014, indicating the effectiveness of implemented emergency risk management strategies. Crucially, this study also incorporated a risk characterization step by estimating the potential incidence of hepatocellular carcinoma (HCC) attributable to AFM_1_ exposure in the adult population. This early application of a disease burden framework represented a pioneering effort in Serbia to quantify the broader public health implications of mycotoxin exposure.

A major methodological advancement came with the study by Milićević et al. [10], which for the first time used nationally representative food consumption data for children aged 1–9 years, collected in accordance with EFSA’s EU Menu methodology. By applying these harmonized intake data to AFM1 occurrence in milk, the study achieved a high-resolution exposure assessment that aligned with EU risk assessment standards. Risk characterization was conducted using the MOE model, providing robust estimates for potential adverse health outcomes in one of the most vulnerable population segments.

The most technically advanced exposure studies include those by Udovički et al. [24] and Djekić et al. [45]. These studies employed probabilistic Monte Carlo simulations in line with EFSA guidelines, allowing for the incorporation of individual variability in consumption behavior, contamination levels, and body weight. Particularly noteworthy is the study by Udovički et al. [24], which utilized second-order Monte Carlo simulations to distinguish natural variability from input parameter uncertainty. This advanced modeling technique enabled a more refined and realistic characterization of HCC risk on an annual basis, especially for high-exposure subgroups such as children in the upper percentiles of milk consumption. Their findings revealed that while mean exposures remained within acceptable limits, a fraction of the population, particularly children, fell below MOE safety thresholds, warranting focused mitigation efforts.

Collectively, these studies illustrate several important outcomes:

(i) Children consistently appear as the most vulnerable demographic group due to higher milk consumption relative to body weight.

(ii) Harmonization with EFSA methodologies ensures international comparability and regulatory alignment. 

(iii) Pasteurized milk and yogurt are identified as the primary sources of AFM1 exposure in Serbia’s adult population. 

(iv) Seasonal and climatic variability continue to be key determinants of contamination levels, underscoring the need for dynamic and responsive control systems.

However, it is important to emphasize that comparing human health risk assessments of AFM_1_ exposure across studies is not straightforward. Methodologies for exposure estimation, population risk characterization, and the calculation of estimated liver cancer risk due to milk consumption vary significantly among studies. This variability limits the ability to identify reliable trends, establish links between environmental conditions and other factors affecting AFM_1_ contamination or exposure, or draw direct comparisons across studies. Given that AFs are recognized causes of chronic non-communicable diseases such as liver cancer, risk assessments must be conducted through protocols designed to evaluate long-term exposure-outcome relationships. Such assessments should follow EFSA’s recommended [38] frameworks to ensure validity, comparability, and policy relevance. Moreover, the integrated risk characterization frameworks adopted in these studies, especially those linking AFM_1_ exposure with HCC burden estimates, highlight the importance of viewing food safety not only through the lens of regulatory compliance but also in terms of long-term public health outcomes. The work of Milićević et al. [10], Udovički et al. [24] Djekić et al. [42] and Krstović et al. [45] collectively provides the empirical and methodological foundation for risk-based prioritization strategies, supporting the broader objective of transitioning Serbia’s food safety system toward a One Health and prevention-oriented paradigm [74].

Overall, the findings from AFM_1_ occurrence and dietary exposure studies provide a robust scientific basis for strengthening risk-based food safety governance in Serbia. While much of the existing data has been generated through independent, investigator-led research rather than through a centralized and institutionalized monitoring framework, these studies consistently highlight AFM_1_ contamination as a persistent food safety concern with significant public health implications. To operationalize their strategic potential in evidence-based risk management, the results of these studies should be systematically incorporated into structured food safety prioritization processes, such as the identification of high-risk contaminants, vulnerable population groups, and critical seasonal and regional patterns of exposure [25,46]. These processes should be aligned with broader public health functions, including the analysis of risk factors and the definition of national health program priorities, thereby ensuring that food safety interventions are integrated into overarching population health strategies. This transition from reactive to proactive governance necessitates structured coordination among food safety authorities, scientific institutions, and inspection bodies [32].

### 3.3. Integrating Risk Ranking into Targeted Food Safety Governance

The persistence of AFM_1_ contamination in Serbia’s dairy sector underscores the urgent need for structured, risk-based approaches to food safety governance. Applying the FAO’s [25] and EFSA [26] risk-ranking framework, AFM_1_ emerges as a high-priority hazard due to its widespread occurrence, toxicological potency, and particularly severe impact on children, who are more vulnerable due to higher milk consumption relative to body weight [30]. Effective risk prioritization ensures that mitigation efforts move beyond end-product testing and toward proactive control measures, especially at the level of feed safety and primary production [75]. However, the current structure of Serbia’s food safety system, characterized by a distribution of responsibilities across several competent authorities and private sector actors, requires more structured and operationalized cross-sectoral coordination—particularly through formalized data-sharing mechanisms, harmonized inspection and monitoring protocols, and coordinated feed–milk surveillance strategies—to effectively translate scientific insights into timely and impactful action. This need is underscored by findings of the State Audit Institution [29], which reported that key inter-institutional coordination bodies and working groups do not function effectively, limiting the system’s ability to implement integrated, risk-based decision-making. Recognizing AFM_1_ as a permanent strategic priority within Serbia’s food safety agenda would improve regulatory coherence, enhance institutional responsiveness, and strengthen public health protection. The following subsections elaborate on two essential pillars of this risk-based strategy:

#### 3.3.1. Integration of Risk Ranking into Control Programs

Operationalizing a risk-based approach requires Serbia to establish regular, institutionalized hazard ranking exercises within its national food control system. The institutionalized risk-ranking process would allow for direct comparison of AFM_1_ with other hazards based on three key dimensions: hazard severity, population vulnerability, and exposure level. To ensure effective implementation, a multisectoral working group should be designated, comprising representatives from the Ministry of Agriculture, Forestry and Water Management, the Ministry of Health, the National Reference Laboratory, internationally accredited laboratories (e.g., ISO/IEC 17025), academic institutions, and food business operators (FBOs). Such a coordinated, multisectoral mechanism is particularly important in Serbia’s context, where effective implementation of risk-based approaches depends on clear operational procedures, seamless collaboration among competent authorities, and consistent exchange of relevant data.

**Figure 4:**
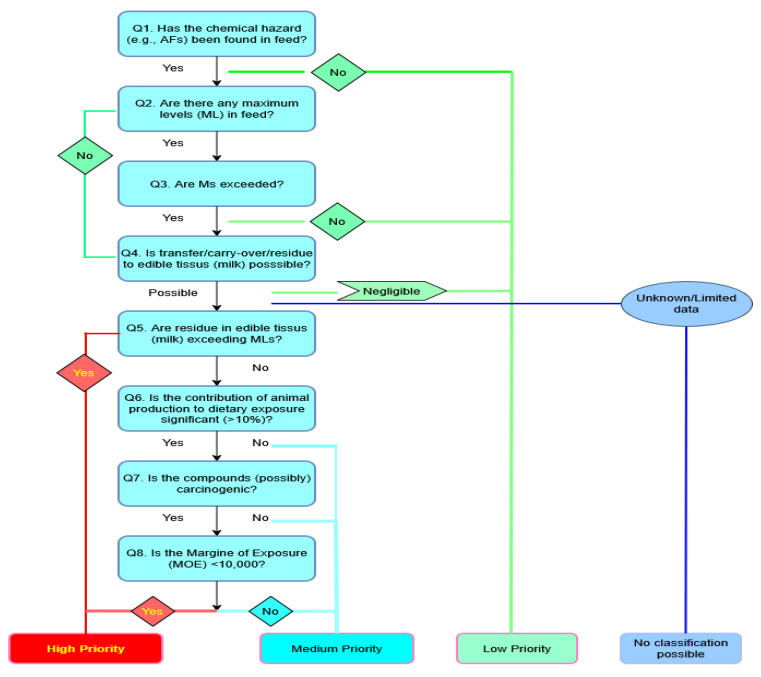
Decision tree for risk ranking and prioritization of AFM1 in the dairy chain for monitoring and control (adopted by [76]).

FBOs, given their legal responsibility for food safety and quality assurance, play a central role in translating risk-ranking outcomes into practical preventive and corrective measures. The institutionalized risk-ranking procedures, ideally conducted on an annual or biennial basis, should evaluate AFM_1_ contamination in milk alongside other relevant chemical and microbiological hazards using national monitoring data and epidemiological evidence. Given its well-documented public health significance, particularly its impact on children, AFM_1_ would likely remain among the highest-ranked hazards. This, in turn, would justify enhanced surveillance efforts, region-specific sampling, and targeted interventions within the National Food Safety Plan [27]. This adaptive, risk-based oversight not only improves regulatory efficiency but also enhances transparency and public confidence in food safety measures [76,77]. To complement this adaptive oversight framework, we propose a decision tree for risk prioritization of AFM_1_ in the dairy chain for monitoring and control (Figure 4). 

Flow charts, or decision trees, are increasingly recognized as practical tools for the qualitative ranking of food-related hazards, offering a clear and structured framework for translating hazard identification into actionable priority levels. Their primary purpose is to provide a rapid and transparent screening of hazards, enabling risk managers to assess the likelihood and severity of harm through a sequence of predefined questions. In this context, the proposed flow chart translates the results of hazard ranking into operational priority levels (high, medium, or low) by guiding risk managers through sequential steps (Q1–Q8). It integrates critical elements such as the presence of AFB1 in feed, compliance with maximum or guidance levels, carry-over into milk, and public health impact metrics, including the margin of exposure (MOE) and carcinogenicity. This approach allows for targeted interventions, optimized sampling plans, and early preventive actions to minimize AFM_1_ contamination in milk and ensure that it remains within acceptable safety limits. However, this method strongly depends on expert input to set up the right questions and criteria based on expert judgment and scientific evidence, and it is therefore essential to perform a rigorous expert elicitation study during its development [36]. Once a decision tree is established, it becomes a highly accessible tool for stakeholders, allowing for straightforward classification of hazards into high, medium, and low risks. Nevertheless, effective risk ranking also requires robust institutional infrastructure and ownership. While academic research in Serbia has provided valuable exposure data using EFSA-aligned dietary surveys and probabilistic models, long-term mitigation depends on the capacity of public institutions to implement coordinated, state-led surveillance and response systems.

Embedding AFM1 monitoring into national health programs—particularly through institutions responsible for chronic disease surveillance and dietary risk assessment—would enhance Serbia’s alignment with EU standards and enable a One Health approach that connects animal feed control, milk safety, and public health outcomes [76,78]. This need is underscored by evidence linking AFM1 exposure with HCC. Although HCC is not among the most frequently diagnosed cancers in Serbia, it ranks among the leading causes of cancer-related deaths, suggesting a silent yet serious burden with disproportionate clinical outcomes. This highlights the importance of preventive action and supports the inclusion of AFM_1_ as a priority contaminant in national cancer prevention and food safety policies. To strengthen dynamic, evidence-based governance, Serbia should invest in a centralized digital platform for hazard surveillance that integrates laboratory findings, exposure metrics, and predictive modeling. Leveraging machine learning–based prediction tools, AI-driven image analysis, and blockchain-supported traceability within this platform would provide a robust foundation for early detection of AF risks, real-time decision-making, and more efficient cross-sectoral coordination throughout the feed and food safety system [79,80].

Importantly, risk prioritization must remain iterative and adaptable. Climate change, feed chain shifts, and evolving consumer behaviors continuously alter risk profiles [81]. This will also support the operationalization of a One Health-based model for managing foodborne risks, as further elaborated in the following section.

#### 3.3.2. Targeting High-Risk Hotspots and Populations 

**Figure 5:**
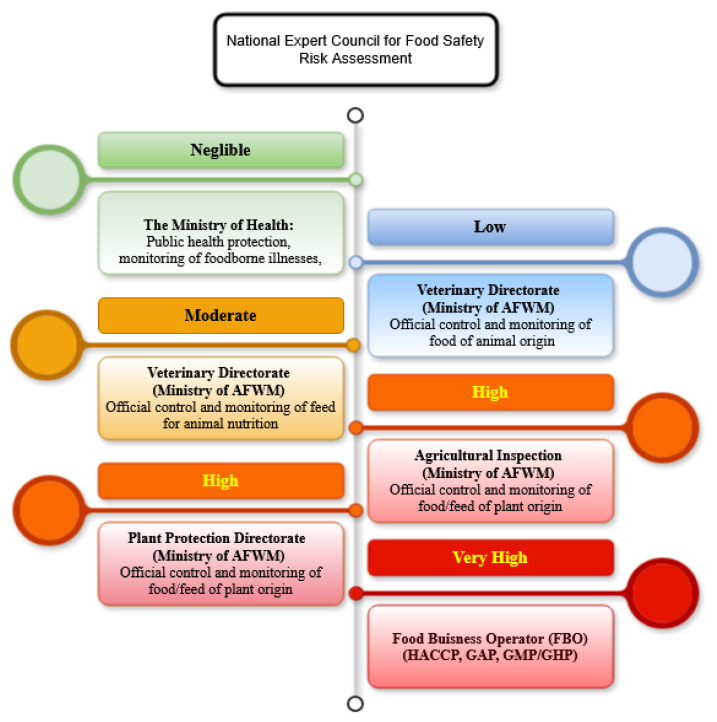
Risk-based ranking, prioritization, and allocation of responsibilities for control of AFs along the dairy chain (Ministry of AFWM-.Ministry of Agriculture, Forestry and Water Managemen).

A risk-based approach to food safety implies that monitoring and control efforts should be directed where the likelihood and consequences of contamination are greatest (Figure 5). In the case of AFM_1_ in Serbia, both geographical and temporal patterns of contamination point to specific hotspots and vulnerable groups that require heightened attention. Geographically, the northern regions of Serbia, particularly the autonomous province of Vojvodina, have been repeatedly identified as high-risk zones [21,48,50,51]. These areas are characterized by intensive maize cultivation and dairy production, which together create conditions conducive to AFB_1_ contamination in feed and subsequent AFM_1_ carry-over in milk. Small-scale farms with limited infrastructure and traditional feed storage practices are especially susceptible to mold growth and mycotoxin accumulation [50]. Seasonally, the risk of AFM_1_ contamination in milk is highest during late winter and early spring [19,20,43,59]. This reflects the biological lag between harvest-time AFB_1_ contamination and its appearance in milk via stored feed. Historical surveillance data confirm that major contamination peaks occurred in January–February 2013 and autumn 2015, following aflatoxin-prone maize harvests [21,48]. These seasonal trends highlight the need to increase milk sampling frequency during high-risk periods and to implement real-time predictive triggers, such as drought forecasts or early detection of elevated AFB_1_ levels in the maize crop, to proactively intensify controls [82].

Equally important is the prioritization of vulnerable consumer groups. Children represent the most sensitive subpopulation due to their higher milk consumption per kilogram of body weight compared to adults, resulting in increased relative dietary exposure to AFM_1_ [10,24,45]. This necessitates tighter control of milk products intended for children, particularly in kindergartens, schools, and for infant nutrition. Practical measures include more frequent lot testing of such milk, stricter enforcement of regulatory limits (especially for powdered milk and baby formulas), and reinforced traceability standards [83]. From a One Health perspective, targeting high-risk populations and regions requires integrated action across sectors [84]. Upstream control of AFB_1_ in maize, overseen by phytosanitary authorities, must be aligned with downstream AFM_1_ monitoring by veterinary services. Public health authorities should support these efforts through targeted awareness campaigns aimed at farmers, processors, and consumers, emphasizing the importance of safe feed practices and the selection of tested milk, especially for young children. By strategically concentrating interventions where contamination risk and exposure potential are highest, both geographically and demographically, Serbia can significantly reduce public health risks associated with AFM_1_, optimize resource allocation, and build a more resilient and responsive food safety system.

Figure 5 illustrates the progressive movement of AFs (from AFB1 in contaminated feed to AFM_1_ in dairy products), highlighting critical control points along the milk supply chain. Color intensity reflects the level of risk and the corresponding priority for intervention. The highest risk and intervention priority are found at the level of primary production (e.g., feed contamination), while risk decreases toward the consumer end of the chain, assuming prior controls have been effective.

### 3.4. Policy Alignment and Priority Recommendations

Harmonization of Serbia’s MLs for AFM_1_ in milk with the EU standard of 0.05 µg/kg [16] represents both a public health imperative and a strategic requirement for regulatory alignment. Although Serbia has made progress in adopting risk-based food safety principles, the current ML of 0.25 µg/kg remains less stringent than EU provisions, reflecting a long-standing tension between consumer protection and economic feasibility [17]. Achieving full and stable harmonization would strengthen public health safeguards, enhance transparency, and support Serbia’s progressive integration into EU food safety systems.

Importantly, harmonized standards are also essential for preserving and expanding Serbia’s position in regional dairy markets. Several countries in Southeast Europe have either aligned or are in the process of aligning their national contaminant limits with EU legislation. Maintaining higher national MLs for AFM_1_ increases the risk of trade disruptions, rejection of consignments, and reduced competitiveness of Serbian dairy products in regional export destinations. Thus, regulatory harmonization is not only a domestic public health priority but also a prerequisite for safeguarding market access and ensuring long-term stability of the dairy sector.

However, harmonization alone is insufficient. Achieving and maintaining compliance with stricter AFM_1_ thresholds requires reinforced control measures across the entire milk production chain from farm to fork, with particular emphasis on upstream management of AFB_1_ in animal feed [40,49]. Consistent enforcement of feed regulations, coordinated official controls, and strengthened monitoring capacities are crucial to preventing the entry of contaminated feed into the dairy chain [29]. These efforts must be supported by systematic farmer education, adoption of good agricultural and storage practices, and the use of validated feed management strategies [85,86]. Ensuring effective control throughout this integrated chain requires complementary investments that support timely detection, traceability, and coordinated response mechanisms.

Further progress also depends on sustained investment in technological and analytical capacities. Recent reviews highlight rapid advances in molecular diagnostics, biosensors, chromatographic platforms, and AI-enhanced detection systems, all of which form the basis for modern, responsive mycotoxin surveillance [87]. Translating these technological advances into practical food-safety capacity, however, requires parallel strengthening of national laboratory infrastructure and risk-intelligence systems. This includes expanding the network of accredited laboratories capable of sensitive AFB_1_/AFM_1_ detection, developing predictive tools for AF outbreaks, and deploying digital traceability solutions that enhance early warning and risk communication. Such measures should be embedded within coherent institutional collaboration: the Phytosanitary Directorate, Veterinary Directorate, and Ministry of Health must function as an integrated risk-governance system, ensuring that regulatory decisions are evidence-based and implemented consistently across the feed–milk–public health continuum.

In summary, policy alignment grounded in regulatory harmonization, strengthened feed safety controls, coordinated multisectoral governance, and investment in monitoring and analytical infrastructure, provides the foundation for effectively reducing AFM_1_ exposure, protecting public health, and securing Serbia’s competitiveness within both EU and regional dairy markets.

## 4. Future Directions: Technological and Research Priorities for Risk-Based Food Safety

Building on the findings of AFM_1_ occurrence, exposure assessment, and institutional performance, Serbia’s next strategic step is to transition toward a predictive, data-driven model of food safety governance. Achieving this shift requires the combined application of technological innovation, forward-looking research, and coordinated institutional modernization, enabling national authorities to anticipate contamination trends, optimize control measures, and strengthen multi-hazard risk prioritization. The following sections outline key technological and research priorities necessary to operationalize this transformation.

### 4.1. Predictive Climate Models for Mycotoxin Risk

Develop models that integrate meteorological, agronomic, and remote-sensing data to forecast AFB_1_ contamination in maize and subsequent AFM_1_ transfer into milk. In addition to conventional weather and crop-growth indicators, high-resolution data collected via satellite imagery or drone-based monitoring can significantly enhance the accuracy of these forecasts. Such predictive tools enable early decision-making and support targeted interventions both pre- and post-harvest, allowing timely mitigation during high-risk seasons or climate-stress conditions [27,36,79,80,88,89].

### 4.2. Integrated Digital Infrastructure and Surveillance

Establish a centralized, interoperable digital platform that consolidates data from feed monitoring, milk testing, weather systems, and public health databases. Geospatial visualization, real-time alerts and interoperable access for competent authorities across the feed–food–health interface, such as the veterinary, phytosanitary, and public health authorities [89,90,91].

### 4.3. Artificial Intelligence (AI)-Enabled Risk Prediction and Smart Sampling

Leverage AI techniques, particularly machine learning (ML) models, as analytical engines capable of processing historical and real-time datasets, including climate trends, feed contamination patterns, inspection history, and milk AFM_1_ levels. These models can predict seasonal contamination peaks and identify high-risk farms with greater precision than traditional approaches. By dynamically generating risk scores, AI/ML systems can inform smart sampling strategies, ensuring that inspection resources are directed toward higher-priority nodes within the supply chain [79,80,89,90,91].

### 4.4. Internet of Things (IoT)-Based Monitoring and Early Warning Systems

Deploy IoT sensors across the dairy chain to track key indicators such as temperature, humidity, and CO_2_ in feed storage, as well as AFM_1_ levels in raw milk at collection points. These sensor networks can automate early warnings and trigger rapid mitigation actions, particularly during drought-driven contamination cycles [79,89,91]. When integrated with AI/ML models predicting seasonal contamination peaks, IoT systems enhance the accuracy and timeliness of early warning mechanisms, enabling dynamic, data-driven adjustments to monitoring and control strategies. 

### 4.5. Multi-Hazard Risk Ranking and Prioritization Tools

Design and pilot risk prioritization tools, such as MCDA and semi-quantitative scoring systems, tailored to Serbian data. These tools should support comparison across chemical, biological, and emerging food safety hazards [27,90,92].

### 4.6. Institutional Readiness and Capacity Building

Assess the preparedness of regulatory agencies, laboratories, and inspection bodies for implementing AI- and IoT-enhanced systems. Strengthen capacity through targeted training programs that enable stakeholders to interpret, trust, and operationalize digital outputs [36,79,80,90,91].

### 4.7. Translational Research and Public Communication

Translate scientific findings into operational protocols, policies, and updated regulatory frameworks. Concurrently, develop evidence-based risk communication strategies to increase transparency, build public trust, and support behavioral change among farmers and consumers.

By aligning these technological and research priorities, Serbia can build a resilient, data-driven food safety system capable of adapting to environmental variability, complying with EU standards, and protecting public health in a sustainable and transparent manner.

## 5. Conclusions

This review provides a synthesis of more than a decade of national scientific research following the 2012 AFM1 crisis, demonstrating that AFM_1_ remains a persistent and seasonally amplified food safety challenge in Serbia’s dairy chain. Evidence consistently links climate-driven AFB1 contamination of maize-based feed with predictable peaks of AFM_1_ in milk, underscoring the relevance of this hazard for public health, especially among young children, as the most exposed group.

To address this long-standing issue, Serbia must advance from reactive responses toward structured, risk-based governance. FAO and EFSA risk-ranking frameworks offer a scientifically robust foundation for prioritizing hazards, directing monitoring efforts, and improving targeting of interventions. While the institutional framework formally supports such an approach, its effectiveness depends on the consistent operationalization of risk-based principles across competent authorities, stronger cross-sector coordination, and systematic integration of hazard ranking into national control programs—areas where current practice shows room for improvement.

Looking ahead, digitalized surveillance systems, predictive analytics, and targeted early-warning tools will be essential for timely detection and more efficient allocation of resources. Embedding these capacities into routine food safety governance will enhance the protection of vulnerable populations, support alignment with EU standards, and contribute to a more resilient and transparent dairy safety system capable of responding to climate-driven aflatoxin pressures. 

## Figures and Tables

**Table 1:** Detailed data on AFM1 occurrence in Serbian milk and dairy products produced during the period 2013–2022.

Production Year	Type of Milk and Dairy Products	Np/N (%)	Mean (µg/kg)	Range (µg/kg)	% Above EU MRL ^h^	Analysis Method	Reference
2013	Heat-treated and raw milk	47/50 (94.0)	0.30	<LOD–1.44	76.0	UHPLC/HESI-MS/MS	[15]
2013	Heat-treated and raw milk	148/150 (98.7)	0.21	0.010–1.20	86.0	ELISA	[56]
	Goat milk	8/10 (80.0)	0.08	0.008–0.24	/		
	Donkey milk	3/5 (60.0)	0.02	0.005–0.03	/		
	Breast milk	6/10 (60.0)	0.01	0.006–0.02	/		
2013	White and hard cheese	29/54 (53.7)	0.08–0.64	0.08–2.23	13.0 ^a^	UHPLC-MS/MS	[44]
2013	Raw milk	934/2045 (45.7)	/	0.005–1.25	/	ELISA/UPLC/MS-MS	[54]
2013/2014	Raw milk	540/678 (79.6) ^b^	0.282	<0.025–>1.0	56.3	ELISA	[57]
	Heat-treated milk	317/438 (72.4) ^b^	0.09	<0.025–>1.0	32.6		
	Milk powder	22/67 (32.8) ^b^	0.847	<0.025–>1.0	25.3		
	Yogurt	42/56 (75.0) ^b^	0.081	<0.025–0.5	39.2		
	Ice cream	14/21 (66.6) ^b^	0.071	<0.025–0.5	52.3		
	Infant formula	2/33 (6.1) ^b^	0.021	<0.025–0.05	0.0		
	White cheese	39/47 (83.0) ^b^	0.146	<0.025–1.0	59.5		
	Hard cheese	21/27 (77.8) ^b^	0.379	<0.025–>1.0	59.2		
	Other	44/71 (62.0) ^b^	0.082	<0.025–1.0	39.4		
2013/2014	Heat-treated milk (2013)	20/20 (100.0)	0.133	0.024–0.319	75.0	HPLC-FLD	[58]
	Heat-treated milk (2014)	54/60 (90.0)	0.026	<LOD–0.104	10.0		
	Infant formula	1/21 (5.0)	0.02	0.02	7.0		
2015	Raw milk (from farms)	36/42 (85.7)	0.008	0.005–0.04	0	ELISA	[50]
	Raw milk (from individual producers)	32/38 (84.2)	0.23	0.006–0.864	63.2	HPLC	
2015	Milk and milk drinks	81/358 (22.6) ^b^	0.018	0.005–0.310	5.9	ELISA	[59]
	Fermented dairy products	63/302 (20.9) ^b^	0.019	0.005–0.320	2.6		
2015	Raw milk	503/1207 (41.7) ^b^	0.037	0.005–0.263	29.3	ELISA	[60]
	Dairy products	236/997 (23.7) ^b^	0.019	0.005–0.320	4.2		
2015–2016	Heat-treated milk	1117/1233 (90.6)	0.035	0.005–0.28	17.3	ELISA	[61]
	Infant formula	23/349 (6.6)	0.011	0.005–0.017	0.2		
	Milk powder	25/94 (26.6)	0.018	0.005–0.035	0		
	Dairy drinks	13/58 (22.4)	0.034	0.005–0.147	5.2		
2015/2016	Raw milk (2015)	984/1408 (70)	0.076	0.005–1.26	30.0	ELISA/LC-MS/MS	[18]
	Raw milk (2016)	3094/3646 (85)	0.069	0.005–1.10	31.0		
2013/2016	Heat-treated and raw milk	342/423 (80.9)	0.216	0.005–5.078	49.1	HPLC-FLD	[51]
2015/2018	Raw milk	236/385 (61.3) ^b^	0.142	<0.025–0.63 ^c^	46.2	ELISA	[45]
	Various dairy products	500/556 (10.1) ^b^	0.016	<0.025–0.029 ^c^	1.3		
2018/2019	Milk and whey	0/30 (0.0)	/	/	0.0	ELISA	[43]
	Goat milk and whey	0/30 (0.0)	/	/	0.0		
2019–2020	Cheese (soft, semi-hard and hard/cow, goat and sheep) ^e^	42/60 (70)	70–77 ^d^	26–591	/	ELISA/HPLC-FLD	[23]
2021	Raw and heat-treated milk	51/284 (17.9)		0.02–0.26	3.1	ELISA	[55]
	Cheese	15/20 (75)		0.15–0.46	/		
2015/2022	Raw milk	6341/8181 (77.5)	0.026–0.027 ^d^	0.005–0.554	16.4	ELISA	[24]
	Heat-treated milk	1541/1864 (82.7)	0.018–0.019 ^d^	0.005–0.193	4.4		
	Yogurt	1457/1985 (73.4)	0.018–0.020 ^d^	0.010–0.137	3.2		
	Chocolate milk and milkshakes	402/507 (79.3)	0.009–0.010 ^d^	0.005–0.045	0.0		
	Fermented (sour) milk	320/409 (78.2)	0.018–0.020 ^d^	0.010–0.104	0.1		
	Fermented cream	243/449 (54)	0.012–0.017 ^d^	0.010–0.294	1.6		
	Butter and clotted cream	3/141 (2.1)	0.002	0.109–0.128	2.1		
	Cheese	58/73 (79.5)	0.123–0.140 ^d^	0.050–0.390	8.6 ^a^		
	Cream	21/50 (42.0)	0.007–0.013 ^d^	0.011–0.033	0.0		
	Various products	428/586 (73.0)	0.018–0.019 ^d^	0.005–0.131	6.8		
2022	Milk and dairy products (cow and goat)	87/107 (81.3)	0.007–0.202 ^f^	0.005–0.246	81.1 ^g^	ELISA	[21]
2021–2025	Raw milk	907 (70.1)	0.086–0.263	0.044–0.302	70.1	ELISA	[42]

This table includes data from both occurrence and exposure studies, with overlapping years and institutions; therefore, some sample repetition may be present. Np—number of positive samples; N—number of analyzed samples; (%)—percentage of positive samples. LOD—Limit of Detection. ^a^—based on the value of 0.25 μg/kg (Škrbić et al., 2015) [44]. ^b^—calculated with negative values reported as ≤0.025 μg/kg. ^c^—95th percentile. ^d^—lower to upper bound range (mean is obtained by replacement of non-detects by zero or LOD). ^e^—study included imported milk products. ^f^—depending on the product. ^g^—of cow milk samples. ^h^—EU maximum level (ML) for AFM1 in milk: 0.05 µg/kg (Commission Regulation (EU) 2023/915). Current Serbian ML for raw milk: 0.25 µg/kg.

**Table 2:** A detailed overview of exposure and risk assessment studies on AFM1 intake in Serbia.

Type of Product	Assessment Method	Type of Food Intake Data	Body Weight Data	Population Group	Average EDI (ng/kg bw/day)	Risk Characterization			Reference
						HI ^a^	MoE ^b^	HCC ^c^	
Milk	Deterministic	Serbian market basket	Average of 60 kg	Adults	0.503–1.420	2.5–7.1	/	/	[15]
Milk	Deterministic	Milk intake questionnaire	Obtained from questionnaires	1–5 years	6.45 (male) 6.26 (female)	/	/	/	[56]
				5–15 years	2.34 (male) 1.86 (female)	/	/	/	
				15–25 years	1.26 (male) 0.42 (female)	/	/	/	
				25–55 years	0.49 (male) 0.56 (female)	/	/	/	
				>55 years	0.51 (male) 0.69 (female)	/	/	/	
Milk	Deterministic	National Statistical Office data	Average of 60 kg	Adults	0.30 (2013) 0.06 (2014)	/	/	0.004 ^d^	[58]
Milk	Deterministic	[56]	[56]	Adult males	0.02	/	/	/	[18]
				Adult females	0.18	/	/	/	
Milk ^e^	Deterministic	[56]	[56]	1–4 years	1.04 (male) 1.01 (female)	5.18 (male) 5.06 (female)	/	0.088 (male) 0.084 (female)	[61]
				5–15 years	0.38 (male) 0.30 (female)	1.82 (male) 1.43 (female)	/		
				16–25 years	0.21 (male) 0.07 (female)	0.98 (male) 0.33 (female)	/		
				26–55 years	0.08 (male) 0.09 (female)	0.38 (male) 0.43 (female)	/		
				>55 years	0.09 (male) 0.12 (female)	0.41 (male) 0.53 (female)	/		
Milk and yogurt	Probabilistic (Monte Carlo simulation)	1-day and 7-day recall questionnaires	Obtained from questionnaires	Student population	1.238–2.674	6.2–13.4	213.2–460.4	0.0017–0.0047	[62]
Milk and dairy products	Probabilistic (Monte Carlo simulation)	1-day and 7-day recall questionnaires	Obtained from questionnaires	Adults	0.076–0.062	/	/	/	[45]
Milk and dairy products	Deterministic	National Food Consumption Survey	Obtained from survey	Toddlers (1–3 years)	0.340–0.475 (male) ^f^ 0.365–0.501 (female) ^f^	/	>10,000	0.00066–0.00257 (male) ^h^ 0.00071–0.00271 (female) ^h^	[10]
				Children (3–9 years)	0.201–0.277 (male) ^f^ 0.190–0.262 (female) ^f^	/	>10,000	0.00069–0.00150 (male) ^h^ 0.00037–0.00142 (female) ^h^	
Cheese ^g^	Deterministic	National Statistical Office data	Institute of Public Health data	Preschool children	0.165–0.182 ^f^	/	>10,000	0.00029–0.00160 ^h^	[23]
				7–10 years	0.100–0.109 ^f^	/	>10,000	0.00017–0.00096 ^h^	
				11–14 years	0.064–0.070 ^f^	/	>10,000	0.00011–0.00062 ^h^	
				15+	0.043–0.048 ^f^	/	>10,000	0.00008–0.00042 ^h^	
Milk and dairy products	Probabilistic (2nd order Monte Carlo simulation)	FFQ questionnaire	Obtained from questionnaires	Adult females	0.161	/	>10,000	0.0003–0.0009 ^h^	[24]
				Adult males	0.126	/	>10,000	0.0003–0.0007 ^h^	
				Adolescents	0.183	/	>10,000	0.0004–0.0010 ^h^	
				Children	0.336	/	>10,000	0.0007–0.0019 ^h^	

^a^—the HI higher than 1 indicates risk to consumers. ^b^—when the MoE value is >10,000, it is considered that there is a low risk of a negative impact on public health. ^c^—cases per year per 100,000 people. ^d^—cases for the whole adult population. ^e^—presented data for the total 2015/2106 heat-treated samples exposure calculation. ^f^—based on the lower to upper bound AFM1 concentration range. ^g^—based on the consumption of both domestic and imported cheeses. ^h^—range is based on different cancer potency estimates. HI—hazard index; MoE—Margin of exposure; HCC—Hepatocellular carcinoma.

## Data Availability

No new data were created or analyzed in this study.

## Abbreviations

The following abbreviations are used in this manuscript:

AFM1	Aflatoxin M1
AFs	Aflatoxins
AFB1	Aflatoxin B1
AI	Artificial intelligence
BMDL	Benchmark dose levels
COR	Carry-Over Rate
DALYs	Disability-Adjusted Life Years
FAO	Food and Agriculture Organization
FBOs	Food Business Operators
EDI	Estimated Daily Intake
EFSA	European Food Safety Authority
ELISA	Enzyme-Linked Immunosorbent Assay
EU	European Union
HACCP	Hazard analysis and critical control points
HBGVs	Health-based guidance values
HCC	Hepatocellular carcinoma
HI	Hazard Index
HPLC	High-performance liquid chromatography
IARC	International Agency for Research on Cancer
IoT	Internet of Things
LC-MS/MS	Liquid Chromatography-Tandem Mass Spectrometry
ML	Machine learning
MRLs	Maximum residue limits
MCDA	Multi-Criteria Decision Analysis
MOE	Margin of Exposure
UHT	Ultra-high temperature processing
YLL	Years of Life Lost
YLD	Years Lived with Disability
QALYs,	Quality-Adjusted Life Years
WHO	World Health Organization

## References

[1] Adjovi Y.C.S., Fossou J.P.M., Ahehehinnou U.H. (2025). A Silent Killer in the World: Review on Aspergillus flavus Strains. Toxicon.

[2] IARC (2012). A Review of Human Carcinogens: Chemical Agents and Related Occupations.

[3] Joint FAO/WHO Expert Committee on Food Additives (JECFA) (2017). Evaluation of Certain Contaminants in Food: Eighty-Third Report of the Joint FAO/WHO Expert Committee on Food Additives.

[4] Iqbal S.Z., Jinap S., Pirouz A.A., Faizal A.A. (2015). Aflatoxin M1 in Milk and Dairy Products: Occurrence and Recent Challenges—A Review. Trends Food Sci. Technol..

[5] Liu Z.M., Zhu B.-C., Xue C.-M., Huang X., Gao R.-Y., Liu W.-H., Cheng J.-J., Wang J.-G., Song Z.-B., Chen S.-X. (2025). Bibliometric Analysis of the Correlation between Aflatoxin and Hepatic Carcinoma. Medicine.

[6] Liu Y., Wu F. (2010). Global burden of aflatoxin-induced hepatocellular carcinoma: A risk assessment. Environ. Health Perspect..

[7] Saha Turna N., Comstock S.S., Gangur V., Wu F. (2024). Effects of Aflatoxin on the Immune System: Evidence from Human and Mammalian Animal Research. Crit. Rev. Food Sci. Nutr..

[8] Hsu P., Pokharel A., Scott C.K., Wu F. (2024). Aflatoxin M1 in Milk and Dairy Products: The State of the Evidence for Child Growth Impairment. Food Chem. Toxicol..

[9] Khlangwiset P., Shephard G.S., Wu F. (2011). Aflatoxins and Growth Impairment: A Review. Crit. Rev. Toxicol..

[10] Milićević D.R., Milešević J., Gurinović M., Janković S., Đinović-Stojanović J., Zeković M., Glibetić M. (2021). Dietary Exposure and Risk Assessment of Aflatoxin M1 for Children Aged 1 to 9 Years Old in Serbia. Nutrients.

[11] Roila R., Branciari R., Verdini E., Ranucci D., Valiani A., Pelliccia A., Fioroni L., Pecorelli I. (2021). A Study of the Occurrence of Aflatoxin M1 in Milk Supply Chain over a Seven-Year Period (2014–2020): Human Exposure Assessment and Risk Characterization in the Population of Central Italy. Foods.

[12] Min L., Li D., Tong X., Sun H., Chen W., Wang G., Wang J. (2020). The Challenges of Global Occurrence of Aflatoxin M1 Contamination and the Reduction of Aflatoxin M1 in Milk over the Past Decade. Food Control.

[13] Salari N., Kazeminia M., Vaisi-Raygani A., Jalali R., Mohammadi M. (2020). Aflatoxin M1 in Milk Worldwide from 1988 to 2020: A Systematic Review and Meta-Analysis. J. Food Qual..

[14] Lević J., Gošić-Dondo S., Ivanović D., Stanković S.Ž., Krnjaja V., Bočarov-Stančić A.S., Stepanić A. (2013). An outbreak of *Aspergillus* species in response to environmental conditions in Serbia. Pestic. Phytomed..

[15] Škrbić B., Živančev J., Antić I., Godula M. (2014). Levels of Aflatoxin M1 in Different Types of Milk Collected in Serbia: Assessment of Human and Animal Exposure. Food Control.

[16] European Commission Commission Regulation 2023/915 of 25 April 2023 on Maximum Levels for Certain Contaminants in Food and Repealing Regulation (EC) No 1881/2006. http://data.europa.eu/eli/reg/2023/915/oj.

[17] Jakšić S., Živkov Baloš M., Prodanov Radulović J., Jajić I., Krstović S., Stojanov I., Mašić Z. (2017). Aflatoxin M1 in Milk and Assessing the Possibility of Its Occurrence in Milk Products. Arch. Vet. Med..

[18] Milićević D.R., Spirić D., Radičević T., Velebit B., Stefanović S., Milojević L., Janković S. (2017). A Review of the Current Situation of Aflatoxin M1 in Cow’s Milk in Serbia: Risk Assessment and Regulatory Aspects. Food Addit. Contam. Part A.

[19] Milićević D., Petronijević R., Petrović Z., Djinović-Stojanović J., Jovanović J., Baltić T., Janković S. (2019). Impact of Climate Change on Aflatoxin M1 Contamination of Raw Milk with Special Focus on Climate Conditions in Serbia. J. Sci. Food Agric..

[20] Milićević D., Lakićević B., Petronijević R., Petrović Z., Jovanović J., Stefanović S., Janković S. (2019). Climate Change: Impact on Mycotoxins Incidence and Food Safety. Theory Pract. Meat Process..

[21] Kos J., Radić B., Lešić T., Anić M., Jovanov P., Šarić B., Pleadin J. (2024). Climate Change and Mycotoxin Trends in Serbia and Croatia: A 15-Year Review. Foods.

[22] Republic of Serbia Rulebook on Maximum Levels of Certain Contaminants in Food. https://pravno-informacioni-sistem.rs/eli/rep/sgrs/ministarstva/pravilnik/2024/73/3/reg.

[23] Torović L., Popov N., Živkov-Baloš M., Jakšić S. (2021). Risk Estimates of Hepatocellular Carcinoma in Vojvodina (Serbia) Related to Aflatoxin M1 Contaminated Cheese. J. Food Compos. Anal..

[24] Udovicki B., Keškić T., Aleksić B., Smigić N., Rajković A. (2023). Second Order Probabilistic Assessment of Chronic Dietary Exposure to Aflatoxin M1 in Serbia. Food Chem. Toxicol..

[25] Food and Agriculture Organization of the United Nations, World Health Organization (FAO/WHO) (2020). FAO Guide to Ranking Food Safety Risks at the National Level.

[26] Van der Fels-Klerx H.J., Van Asselt E.D., Raley M., Poulsen M., Korsgaard H., Bredsdorff L., Nauta M., Flari V., D’Agostino M., Coles D. (2015). Critical Review of Methodology and Application of Risk Ranking for Prioritisation of Food and Feed Related Issues, on the Basis of the Size of Anticipated Health Impact. EFSA Supporting Publ..

[27] Van der Fels-Klerx H.J., Adamse P., Punt A., Van Asselt E.D. (2018). Data Analyses and Modelling for Risk Based Monitoring of Mycotoxins in Animal Feed. Toxins.

[28] Hobé R.G., van Asselt E.D., van den Heuvel L., Hoek-van den Hil E.F., van der Fels-Klerx H.J. (2023). Methodology for risk-based monitoring of contaminants in food: A case study in cereals and fish. Food Res Int..

[29] State Audit Institution of the Republic of Serbia (2023). Food Safety in the Republic of Serbia: Audit Report.

[30] Milićević D.R., Udovički B., Šuša A., Rajković A., Pleadin J. (2025). Risk Ranking and Risk-Based Prioritisation of Aflatoxin M1 in Milk and Dairy Products Supporting Risk-Based Monitoring and Food Safety Governance. Food Addit. Contam. Part A.

[31] Svendsen C., Mathisen G.H., Vist G.E., Husøy T., Ames H.M., Beronius A., Whaley P. (2024). Cross-Mapping of Terms Used in Chemical Risk Assessment with Those Used in Systematic Review: Research Protocol. Evidence-Based Toxicology.

[32] Food and Agriculture Organization of the United Nations, World Health Organization (FAO/WHO) (2019). Food Control System Assessment Tool: Introduction and Glossary. https://www.fao.org/3/ca5339en/CA5339EN.pdf.

[33] Pires S.M., Redondo H.G., Pessoa J., Jakobsen L.S., Thomsen S.T. (2024). Risk Ranking of Foodborne Diseases in Denmark: Reflections on a National Burden of Disease Study. Food Control.

[34] U.S. EPA (2019). Guidelines for Human Exposure Assessment.

[35] Lu H. (2022). Comparison of Aflatoxin-Induced Hepatocellular Carcinoma in the United States and China. Ph.D. Thesis.

[36] Van der Fels-Klerx H.J., Van Asselt E.D., Raley M., Poulsen M., Korsgaard H., Bredsdorff L., Nauta M., D’Agostino M., Coles D., Marvin H.J.P. (2018). Critical Review of Methods for Risk Ranking of Food-Related Hazards, Based on Risks for Human Health. Crit. Rev. Food Sci. Nutr..

[37] Svendsen C., Amlund H., Carlsen M.H., Eriksen G.S., Husøy T., Lillegaard I.T.L., Mathisen G.H., Medin A.C., Ørnsrud R., Agdestein A. (2022). Food and Chemical Substances Relevant for Monitoring. Report from the Scientific Steering Committee of the Norwegian Scientific Committee for Food and Environment.

[38] Schrenk D., Bignami M., Bodin L., Chipman J.K., Del Mazo J., Grasl-Kraupp B., Hogstrand C., Hoogenboom L., Leblanc J., EFSA Panel on Contaminants in the Food Chain (CONTAM) (2020). Risk Assessment of Aflatoxins in Food. EFSA J..

[39] Fink-Gremmels J. (2008). Mycotoxins in Cattle Feeds and Carry-Over to Dairy Milk: A Review. Food Addit. Contam..

[40] Zentai A., Jóźwiak Á., Süth M., Farkas Z. (2023). Carry-Over of Aflatoxin B1 from Feed to Cow Milk—A Review. Toxins.

[41] Transfer Model Aflatoxin B1—Dairy Cow, Version 1.1. http://www.feedfoodtransfer.nl.

[42] Krstović S., Jakšić S., Miljanić J., Iličić B., Živkov Baloš M., Guljaš D., Damjanović M., Jajić I. (2025). Annual and Seasonal Variations in Aflatoxin M1 in Milk: Updated Health Risk Assessment in Serbia. Toxins.

[43] Milićević D., Janković S., Murić K., Petrović Z., Petronijević R., Rašeta M., Đinović-Stojanović J. (2019). Safety of Milk and Whey from Zlatibor Region in Relation to Aflatoxin M1 Contamination: A Seasonal Study. IOP Conf. Ser. Earth Environ. Sci..

[44] Škrbić B., Antić I., Živančev J. (2015). Presence of Aflatoxin M1 in White and Hard Cheese Samples from Serbia. Food Control.

[45] Djekic I., Petrovic J., Jovetic M., Redzepovic-Djordjevic A., Stulic M., Lorenzo J.M., Tomasevic I. (2020). Aflatoxins in Milk and Dairy Products: Occurrence and Exposure Assessment for the Serbian Population. Appl. Sci..

[46] Food and Agriculture Organization of the United Nations, World Health Organization (FAO/WHO) (2023). General Principles of Food Hygiene. Codex Alimentarius Code of Practice, No. CXC 1-1969.

[47] Chen Z., Mullins C.D., Novak P. (2015). The Evolution of the Disability-Adjusted Life Year (DALY). Value Health.

[48] Kos J., Radić B., Radović R., Šarić B., Jovanov P., Šarić L. (2024). Aflatoxins in Maize, Milk and Dairy Products from Serbia. Food Addit. Contam. Part B.

[49] Ferrari L., Rizzi N., Grandi E., Clerici E., Tirloni E., Stella S., Bernardi C.E.M., Pinotti L. (2023). Compliance between Food and Feed Safety: Eight-Year Survey (2013–2021) of Aflatoxin M1 in Raw Milk and Aflatoxin B1 in Feed in Northern Italy. Toxins.

[50] Polovinski Horvatović M., Glamočić D., Jajić I., Krstović S., Guljaš D., Gjorgjievski S. (2016). Aflatoxin M1 in Raw Milk in the Region of Vojvodina. Mljekarstvo.

[51] Jajić I., Glamočić D., Krstović S., Horvatović M.P. (2018). Aflatoxin M1 Occurrence in Serbian Milk and Its Impact on Legislative. J. Hellenic Vet. Med. Soc..

[52] Republic of Serbia (2025). Law on Official Controls of the Republic of Serbia [Unofficial English translation]. https://minpolj.gov.rs/zakoni/.

[53] Milićević D., Udovički B., Petrović Z., Janković S., Radulović S., Gurinović M., Rajković A. (2020). Current Status of Mycotoxin Contamination of Food and Feeds and Associated Public Health Risk in Serbia. Meat Technol..

[54] Spirić D.M., Đinović J.M., Janković V.V., Velebit B.M., Radičević T.M., Stefanović S.M., Janković S.D. (2015). Study of Aflatoxins Incidence in Cow Feed and Milk in Serbia during 2013. Hem. Ind..

[55] Jauković M.M., Rokvić N.I., Vuksan A.D. (2024). Recent Aflatoxin Levels in Maize, Feed Mixtures, Milk and Cheese in Serbia. Zb. Matice Srp. Prir. Nauke.

[56] Kos J., Lević J., Đuragić O., Kokić B., Miladinović I. (2014). Occurrence and Estimation of Aflatoxin M1 Exposure in Milk in Serbia. Food Control.

[57] Tomašević I., Petrović J., Jovetić M., Raičević S., Milojević M., Miočinović J. (2015). Two Year Survey on the Occurrence and Seasonal Variation of Aflatoxin M1 in Milk and Milk Products in Serbia. Food Control.

[58] Torović L. (2015). Aflatoxin M1 in Processed Milk and Infant Formulae and Corresponding Exposure of Adult Population in Serbia in 2013–2014. Food Addit. Contam. Part B.

[59] Keškić T., Miočinović J., Kos A., Gavrić M., Miloradović Z., Puđa P. (2016). Seasonal Variation of Aflatoxin M1 in Dairy Products during 2015 in Serbia. In Proceedings of Second International Symposium of Veterinary Medicine.

[60] Miočinović J., Keškić T., Miloradović Z., Kos A., Tomašević I., Puđa P. (2017). The Aflatoxin M1 Crisis in the Serbian Dairy Sector: The Year After. Food Addit. Contam. Part B.

[61] Milićević D., Spirić D., Janković S., Velebit B., Radičević T., Petrović Z., Stefanović S. (2017). Aflatoxin M1 in Processed Milk: Occurrence and Seasonal Variation with an Emphasis on Risk Assessment of Human Exposure in Serbia. IOP Conf. Ser. Earth Environ. Sci..

[62] Udovicki B., Audenaert K., De Saeger S., Rajkovic A. (2018). Overview on the Mycotoxins Incidence in Serbia in the Period 2004–2016. Toxins.

[63] Bilandžić N., Varenina I., Solomun Kolanović B., Božić Đ., Đokić M., Sedak M., Tanković S., Potočnjak D., Cvetnić Ž. (2015). Monitoring of Aflatoxin M1 in Raw Milk during Four Seasons in Croatia. Food Control.

[64] Bilandžić N., Varga I., Varenina I., Solomun Kolanović B., Božić Luburić Đ., Đokić M., Cvetnić Ž. (2022). Seasonal Occurrence of Aflatoxin M1 in Raw Milk during a Five-Year Period in Croatia: Dietary Exposure and Risk Assessment. Foods.

[65] Pleadin J., Vulić A., Perši N., Škrivanko M., Čapek B., Cvetnić Ž. (2014). Aflatoxin B1 Occurrence in Maize Sampled from Croatian Farms and Feed Factories during 2013. Food Control.

[66] Topi D., Spahiu J., Rexhepi A., Marku N. (2022). Two-Year Survey of Aflatoxin M1 in Milk Marketed in Albania, and Human Exposure Assessment. Food Control.

[67] Buzás H., Szabó-Sárvári L.C., Szabó K., Nagy-Kovács K., Bukovics S., Süle J., Kovács A.J. (2023). Aflatoxin M1 Detection in Raw Milk and Drinking Milk in Hungary by ELISA: A One-Year Survey. J. Food Compos. Anal..

[68] Rama A., Latifi F., Bajraktari D., Ramadani N. (2015). Assessment of Aflatoxin M1 Levels in Pasteurized and UHT Milk Consumed in Prishtina, Kosovo. Food Control.

[69] Dimitrieska-Stojković E., Stojanovska-Dimzoska B., Ilievska G., Uzunov R., Stojković G., Hajrulai-Musliu Z., Jankuloski D. (2016). Assessment of Aflatoxin Contamination in Raw Milk and Feed in Macedonia during 2013. Food Control.

[70] Făţ A., Dan S.D., Tăbăran A., Reget O., Mikle D., Mihaiu R., Mihaiu M. (2018). The Incidence of Aflatoxin M1 in Milk Produced in a Regional Surveillance System Between 2013 and 2017. Bull. Univ. Agric. Sci. Vet. Med. Cluj-Napoca Vet. Med..

[71] Turkoglu C., Keyvan E. (2019). Determination of Aflatoxin M1 and Ochratoxin A in Raw, Pasteurized and UHT Milk in Turkey. Acta Sci. Vet..

[72] Panara A., Katsa M., Kostakis M., Bizani E., Thomaidis N.S. (2022). Monitoring of Aflatoxin M1 in Various Origins Greek Milk Samples Using Liquid Chromatography Tandem Mass Spectrometry. Separations.

[73] Nesic K., Milicevic D., Nesic V., Ivanovic S.D. (2015). Mycotoxins as One of the Foodborne Risks Most Susceptible to Climatic Change. Proceedings of the 58th International Meat Industry Conference (MEATCON2015), Zlatibor, Serbia, 4-7 October 2015.

[74] Garcia S.N., Osburn B.I., Jay-Russell M.T. (2020). One Health for Food Safety, Food Security, and Sustainable Food Production. Front. Sustain. Food Syst..

[75] Ji X., Zhou Y., Xiao Y., Lyu W., Wang W., Shao K., Yang H. (2024). A Tiered Approach of Hazard-Prioritization and Risk-Ranking for Chemical Hazards in Food Commodities: Application for Selected Mycotoxins. Food Res. Int..

[76] Van Asselt E.D., Noordam M.Y., Pikkemaat M.G., Dorgelo F.O. (2018). Risk-Based Monitoring of Chemical Substances in Food: Prioritization by Decision Trees. Food Control.

[77] Palmont P., Membré J.M., Rivière G., Bemrah N. (2023). Risk ranking of chemical hazards in foods: Comparison of aggregating methods using infant formula as an example. Food Addit. Contam. Part A.

[78] Sampedro F. (2020). Risk Ranking: Moving Towards a Risk-Based Inspection and Surveillance System. Risk Assessment Methods for Biological and Chemical Hazards in Food.

[79] Wang X., Bouzembrak Y., Lansink A.O., Van Der Fels-Klerx H.J. (2022). Application of Machine Learning to the Monitoring and Prediction of Food Safety: A Review. Compr. Rev. Food Sci. Food Saf..

[80] Gamlath C.J., Wu F. (2025). AI and Biotechnology to Combat Aflatoxins: Future Directions for Modern Technologies in Reducing Aflatoxin Risk. Toxins.

[81] Garcia-Cela E., Gasperini A.M. (2024). Climate Change and Mycotoxins: A Growing Food Safety Concern. J. Consum. Prot. Food Saf..

[82] Pavković N., Vranešević M., Bagi F., Iličić R. (2025). Implementation of Biotechnical Measures for the Control and Prevention of Aflatoxin Contamination in Maize Production. Contemp. Agric..

[83] Wu X., Lu Y., Xu H., Lv M., Hu D., He Z., Liu L., Wang Z., Feng Y. (2018). Challenges to improve the safety of dairy products in China. Trends Food Sci. Technol..

[84] Frazzoli C., Gherardi P., Saxena N., Belluzzi G., Mantovani A. (2017). The Hotspot for (Global) One Health in Primary Food Production: Aflatoxin M1 in Dairy Products. Front. Public Health.

[85] Anato A., Headey D., Hirvonen K., Pokharel A., Tessema M., Wu F., Baye K. (2024). Feed Handling Practices, Aflatoxin Awareness and Children’s Milk Consumption in the Sidama Region of Southern Ethiopia. One Health.

[86] Sewunet S.D., Kebede E., Melaku A., Assefa A.Y., Alebie A., Assefa A., Kenubih A.W. (2024). Dairy Farmers’ Knowledge, Attitudes, and Practices (KAP) Towards Aflatoxin Contamination in Milk and Feeds in Bahir Dar, Ethiopia. Int. J. Microbiol..

[87] Kępka-Borkowska K., Chałaskiewicz K., Ogłuszka M., Borkowski M., Lepczyński A., Pareek C.S., Starzyński R.R., Lichwiarska E., Sultana S., Kalra G. (2025). Current Approaches to Aflatoxin B1 Control in Food and Feed Safety: Detection, Inhibition, and Mitigation. Int. J. Mol. Sci..

[88] Van der Fels-Klerx H.J., Vermeulen L.C., Gavai A.K., Liu C. (2019). Climate Change Impacts on Aflatoxin B1 in Maize and Aflatoxin M1 in Milk: A Case Study of Maize Grown in Eastern Europe and Imported to the Netherlands. PLoS ONE.

[89] Wang X., Liu C., Van der Fels-Klerx H.J. (2022). Regional Prediction of Multi-Mycotoxin Contamination of Wheat in Europe Using Machine Learning. Food Res. Int..

[90] Wang Z., Van der Fels-Klerx H.J., Oude Lansink A.G.J.M. (2020). Optimization of Sampling for Monitoring Chemicals in the Food Supply Chain Using a Risk-Based Approach: The Case of Aflatoxins and Dioxins in the Dutch Dairy Chain. Risk Anal..

[91] Marvin H.J.P., Bouzembrak Y., van der Fels-Klerx H.J., Kempenaar C., Veerkamp R., Chauhan A., Stroosnijder S., Top J., Simsek-Senel G., Vrolijk H. (2022). Digitalisation and Artificial Intelligence for Sustainable Food Systems. Trends Food Sci. Technol..

[92] Focker M., van der Fels-Klerx H.J., Oude Lansink A.G.J.M. (2019). Optimization of the Aflatoxin Monitoring Costs Along the Maize Supply Chain. Risk Anal..

